# Looking outside the box with a pathology aware AI approach for analyzing OCT retinal images in Stargardt disease

**DOI:** 10.1038/s41598-025-85213-w

**Published:** 2025-02-08

**Authors:** Parisa Khateri, Tiana Koottungal, Damon Wong, Rupert W. Strauss, Lucas Janeschitz-Kriegl, Maximilian Pfau, Leopold Schmetterer, Hendrik P. N. Scholl

**Affiliations:** 1 Institute of Molecular and Clinical Ophthalmology Basel, Basel, Switzerland; 2 Singapore Eye Research Institute, Singapore National Eye Centre, Singapore, Singapore; 3 Department of Ophthalmology, Medical University Graz, Graz, Austria; 4 Department of Ophthalmology, University of Basel, Basel, Switzerland; 5 Department of Clinical Pharmacology, Medical University of Vienna, Vienna, Austria; 6 Moorfields Eye Hospital, NHS Foundation Trust and UCL Institute of Ophthalmology, University College London, London, UK; 7 Department of Ophthalmology, University of Bonn, Bonn, Germany; 8 F. Hoffmann-La Roche AG, Basel, Switzerland; 9 Pallas Kliniken AG, Pallas Klinik Zürich, Zürich, Switzerland; 10 European Vision Institute, Basel, Switzerland; 11 SERI-NTU Advanced Ocular Engineering (STANCE) Program, Singapore, Singapore; 12 Ophthalmology and Visual Sciences Academic Clinical Program (Eye ACP), Duke-NUS MedicalSchool, Singapore, Singapore; 13 School of Chemistry, Chemical Engineering and Biotechnology, Nanyang Technological University, Singapore, Singapore; 14 Center for Medical Physics and Biomedical Engineering, Medical University Vienna, Vienna, Austria; 15 Fondation Ophtalmologique Adolphe De Rothschild, Paris, France; 16 Aier Hospital Group, Changsha, People’s Republic of China

**Keywords:** Stargardt Disease, Optical Coherence Tomography, Deep Learning, Retina Segmentation, Pathology-Aware Loss Function, Automated Image Analysis, Diseases, Medical research, Signs and symptoms, Mathematics and computing

## Abstract

Stargardt disease type 1 (STGD1) is a genetic disorder that leads to progressive vision loss, with no approved treatments currently available. The development of effective therapies faces the challenge of identifying appropriate outcome measures that accurately reflect treatment benefits. Optical Coherence Tomography (OCT) provides high-resolution retinal images, serving as a valuable tool for deriving potential outcome measures, such as retinal thickness. However, automated segmentation of OCT images, particularly in regions disrupted by degeneration, remains complex. In this study, we propose a deep learning-based approach that incorporates a pathology-aware loss function to segment retinal sublayers in OCT images from patients with STGD1. This method targets relatively unaffected regions for sublayer segmentation, ensuring accurate boundary delineation in areas with minimal disruption. In severely affected regions, identified by a box detection model, the total retina is segmented as a single layer to avoid errors. Our model significantly outperforms standard models, achieving an average Dice coefficient of $99\%$ for total retina and $93\%$ for retinal sublayers. The most substantial improvement was in the segmentation of the photoreceptor inner segment, with Dice coefficient increasing by $25\%$. This approach provides a balance between granularity and reliability, making it suitable for clinical application in tracking disease progression and evaluating therapeutic efficacy.

## Introduction

Stargardt disease type 1 (STGD1) is a genetic retinal disorder, with a typical onset in childhood or adolescence, which progressively leads to vision loss and results in legal blindness in almost all cases^1,2^. Despite ongoing clinical trials, there are currently no approved therapies^3^. The rarity of the disease and its slow progression pose significant challenges in measuring structural progression, which is critical for understanding the disease’s natural history and developing potential treatments. The ProgStar study, a multicenter longitudinal program conducted throughout the world, has collected extensive data on retinal structure and function, offering a unique opportunity to overcome these challenges^4–6^.

One of the promising endpoints for assessing the structural progression of Stargardt disease is Optical Coherence Tomography (OCT), a key examination method in the ProgStar study^7^. OCT is a non-invasive and patient-friendly procedure with a short acquisition time. It provides high-resolution cross-sectional images of the posterior pole of the retina on microscale, enabling the objective measurement of loss of the photoreceptor layer and retinal pigment epithelium (RPE) atrophy. Notably, OCT can reveal structural changes, such as hyper-reflectivity at the base of the foveal outer nuclear layer, even before onset of symptoms^8,9^.

OCT imaging allows for the quantification of several parameters based on the segmentation of retinal layers, including total retinal thickness and the thickness of sublayers such as inner retina, the outer nuclear layer, photoreceptor inner/outer segments, and the RPE. A recent ProgStar report demonstrated a significant decline of mean thickness and intact area (as defined by thickness value $>0 \upmu m$) of the outer retinal layers, captured through OCT scans of patients with STGD1 over a 24 months period^9^. This supports the potential of OCT imaging and retinal thickness layer as key endpoints for clinical trials aimed at managing the disease progression. However, the experience with manual and semi-automated segmentation of OCT images of patients with STGD1 has demonstrated that this task is complex^10,11^. Continuous segmentation of the photoreceptor inner and outer segment boundaries is particularly challenging due to layer disruptions and the accumulation of hyper-reflective debris due to the disease pathology. Even correcting software errors in semi-automated segmentation is time-consuming, requiring significant resources in terms of time, funding and personnel^12^.

Automatic segmentation of retinal sublayers in OCT images has been widely studied in recent years, employing both classical methods and deep learning-based approaches. The methods usually focus on boundary delineation or pixel-wise classification to identify retinal sublayers. They offer insights into various retinal disorders. However, few studies specifically address the complexities of segmenting OCT images in the context of Stargardt disease, where structural disruptions challenge the segmentation of retinal sublayers. Liu et al. employed an improved Canny operator for automatic segmentation of eleven retinal boundaries in OCT images of healthy subjects and age-related macular degeneration (AMD) patients^13^. Their method showed robustness in handling noise and artifacts, but its reliance on classical edge detection techniques may limit adaptability to the severe disruptions seen in Stargardt disease. Li et al., with DeepRetina, introduced a modified Xception65 architecture^14^ combined with an atrous spatial pyramid pooling^15^ to delineate 10 retinal boundaries in OCT images of healthy subjects. Their approach was notable for its adaptability to high-resolution OCT images and preserving fine-details. UNet architectures^16^ have been widely used for retinal segmentation tasks due to its ability to preserve spatial hierarchies and extract contextual information. For instance, Yojana and Thillai Rani developed a UNet-based model with a ResNet34 encoder-decoder for total retina segmentation in healthy eyes^17^. However, this approach lacks the granularity to address sublayer segmentation. He et al.^18^ introduced a residual UNet consisting of residual blocks with two output branches, combining pixel classification and boundary regression into a single feed-forward operation. Their model was promising in segmenting retinal sublayers in diabetic macular edema (DME) and multiple sclerosis, however, it requires adaptation for the unique disruptions in Stargardt disease. Sousa et al.^19^ integrated UNet and DexiNed^20^ in a two-step pipeline to segment retinal sublayers in OCT images of healthy subjects and AMD patients. Their method effectively captured fine boundary details, but its performance in severely disrupted regions, typical of Stargardt pathology, remains unexplored. Wang et al.^21^ proposed a 3D graph-assisted UNet, integrating a graph-pyramid into a U-shape network for segmentation of OCT volumes in DME and wet AMD patients. Despite its success in segmenting diseased retinas, its dependency on spatial coherence may not generalize well to Stargardt disease, where layer continuity is often lost. More recently, there has been a growing interest in deploying attention-based models and vision transformers for OCT segmentation^22^. Cao et al.^23^ proposed a UNet-based model integrating self-attention mechanisms. A transformer block was added at the encoder’s output to capture long-range dependencies, and spatial attention mechanisms were added in skip connections and upsampling to enhance essential features. Their model was validated on data with DME, myopia, peripapillary atrophy and cataract for nine retinal layers. Similarly Zhang et al. proposed TranSegNet^24^, which combined a lightweight vision transformer with an improved UNet backbone, using multi-head convolutional for global feature extraction. The network was evaluated for the segmentation of retinal OCT images of healthy and DME patients. These models while effective in various retinal pathologies, remain largely unexplored in their utility for the disrupted retinal layers in Stargardt disease.

To our knowledge, very few studies have been conducted on automated OCT segmentation for Stargardt disease to date. Kugelman et al. investigated the use of automated deep learning techniques to segment retinal boundaries in Stargardt patients’ OCT images^25^. However, they focused on segmenting the total retina as a single layer, rather than individual retinal sublayers. Mishra et al. introduced a UNet-based^16^ deep learning model to segment the retina into 11 sublayers in OCT images of Stargardt patients^26^. Another study independently re-segmented a subset of the same dataset which were initially graded for Mishra’s study, and noted reproducibility issues, particularly in grading the outer retinal layers in disrupted areas critical for assessing disease progression^12^. This was attributed to inclusion criteria based on atrophy, which rendered inner-outer segment layers absent in the central subfield. The variability in these measurements raises concerns about the reliability of such complex segmentation methods for consistent disease monitoring. Furthermore, the extensive manual segmentation performed by Mishra et al.^26^ is time-consuming and costly, making it impractical for clinical trials. These approaches face significant limitations: the former lacks granularity, while the latter introduces reproducibility issues, especially in areas where retinal layers are absent due to degeneration.

Addressing these challenges requires establishing reliable ground truth annotations and developing robust, automated segmentation techniques capable of managing the complexities of retinal degeneration while enabling accurate measurements of progressive structural changes of the retina. In this study, we propose a novel deep learning-based approach that incorporates a pathology-aware loss function, targeting relatively unaffected regions where sublayers can still be detected. Retinal sublayer segmentation is performed only in these regions—where disruption is minimal enough to allow for accurate boundary delineation. In regions with severe degeneration, identified by a box detection model, the total retina is segmented as a single layer. This approach prevents the model from attempting to segment layers that are no longer present by distinguishing severely affected regions from relatively unaffected ones, applying tailored segmentation models to each area, thereby reducing errors and enhancing the reliability of structural measurements.

## Methods

### Dataset

The data used for this study were obtained from the prospective arm of the ProgStar study^4^ and from a set of healthy eyes, as described below.

#### ProgStar data

The prospective arm of the ProgStar study provides longitudinal data from 259 patients with molecularly confirmed STGD1, gathered at nine international sites. Details of data acquisition and demographic information of patients have been reported previously^4^.

Almost all patients underwent five study visits every six months, with both eyes examined at each visit. Multiple investigations were performed during these visits, including Spectral Domain OCT (SD-OCT), fundus autofluorescence, and microperimetry. This study specially focused on analyzing SD-OCT images, obtained with the Heidelberg Spectralis (Heidelberg Engineering, Heidelberg, Germany), which consisted of volumetric macular scans with 49 B-scans, each with a resolution of ${1024 \times 496}$ pixels, covering a field of view (FOV) of ${20}^{\circ }$ by ${20}^{\circ }$ (approximately 6 mm $\times$ 6 mm with 2 mm depth).

Quality of SD-OCT images was assessed based on the following criteria: FOV size, image resolution, cut-off artifacts, noise levels, tilting effects, non-uniformity, hyper-reflectivity, reflection artifacts, and eccentric fixation.

Ideally, we would have 2,590 volumes with 126,910 B-scans, but several patients did not have both their eyes examined, did not follow up for some visits, and some B-scans were excluded due to their poor quality. As a result, 105,537 B-scans from the ProgStar dataset were included in the analysis.

In some cases, difficulties with fixation led to the inclusion of the optic nerve in the FOV. These instances were corrected by manually masking the optic nerve. Additionally, due to the registration of data during successive visits, some B-scans were slightly rotated, resulting in oblique cuts through the retina layers; these sections were also masked out.

#### Healthy eyes data

The healthy eyes dataset comprised 100 SD-OCT volumes acquired by the Heidelberg Spectralis system from 100 volunteers at the Department of Ophthalmology, University of Bonn, Germany. The Institutional Review Board (IRB) of the University of Bonn approved the study (ethics approval ID: 191/16). The study adhered to the Declaration of Helsinki and local regulations. Written informed consent was obtained from all participants.

Each SD-OCT volume featured a macular scan with 121 B-scans, each with a resolution of ${768 \times 496}$ pixels, covering an FOV of ${30}^{\circ }$ by ${25}^{\circ }$ (approximately 9 mm $\times$ 7.5 mm with 2 mm depth). To ensure compatibility with the ProgStar dataset, the healthy B-scans were resized using nearest neighbor interpolation to match the matrix size of ProgStar dataset. Additionally, due to the larger FOV in the healthy dataset, some B-scans included the optic nerve, which was masked out whenever it appeared.

### Data preparation

#### Data sampling and stratification

**Fig. 1:**
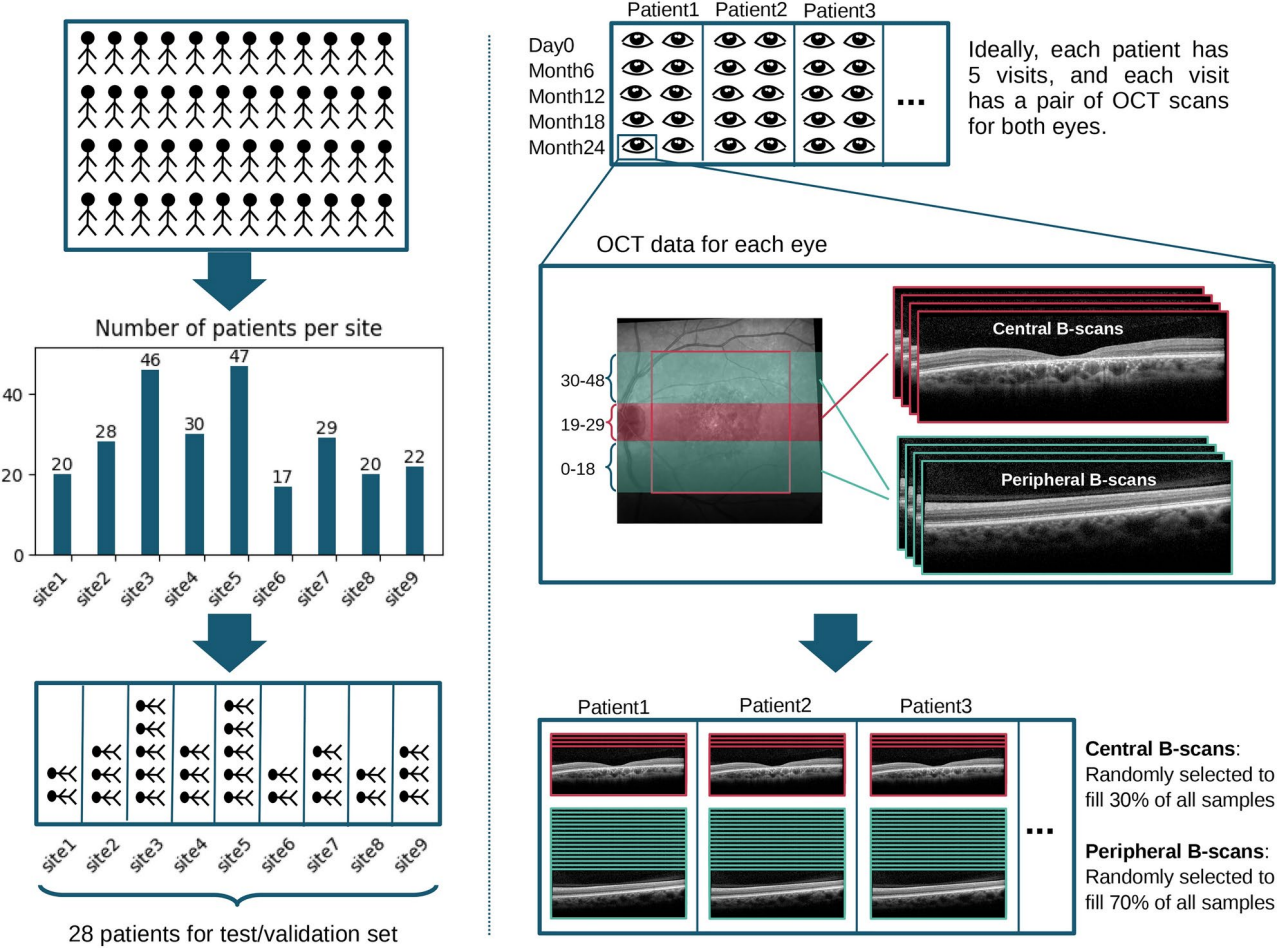
Data sampling and stratification process. Patients from different sites were randomly divided into training, validation, and test sets in an 8:1:1 ratio, ensuring no patient’s data was in more than one set. This resulted in 203 patients in the training set, and 28 patients in each of the validation and test sets. OCT B-scans were divided into central (middle 11 B-scans) and peripheral (remaining 38 B-scans) groups, shuffled across all visits and both eyes. 30% of B-scans were selected from the central group and 70% from the peripheral group.

As detailed above, over $10^5$ B-scans from the ProgStar dataset were available for analysis. Manually annotating such a large number of 2D images is highly time consuming. Therefore, we implemented an iterative framework similar to the active learning method^27^ to minimize the amount of manual annotation required for training the models (see section Iterative Learning). An initial sample for training the deep learning models was stratified and selected as follows (see figure 1):

1. **Site-base stratification**: The ProgStar data were collected from multiple sites, each contributing a different number of patients. Patients from each site were randomly assigned to training, validation and test sets in an 8 : 1 : 1 ratio, ensuring that each site approximately follows this proportion, and no individual patient’s data appears in more than one set. This stratification resulted in 203 patients in the training set and 28 patients in each of the validation and test sets.
2. **B-scan grouping**: B-scans from each SD-OCT volume were divided into central and peripheral groups, with the middle 11 B-scans classified as central and the remaining 38 as peripheral. For each patient, all central and peripheral B-scans, across all visits and both eyes, were grouped and shuffled.
3. **B-scan sampling**: A fixed number of B-scans were sampled from each patient to create the dataset. The sampling ratio was set at $30\%$ central B-scans and $70\%$ peripheral B-scans, ensuring denser sampling from the central region, where the most damage due to the disease typically occurs. The fixed number of B-scans per patient was set to 16 for the validation and test sets, which remain constant during training, and three for the training set, which increases as the training progresses.

This approach resulted in an initial train set of 609 B-scans, and 448 B-scans in both the validation and test sets. A similar procedure was used to sample an equivalent number of images from the healthy dataset. The sampled data were annotated as described in the next section. During the training process, the size of the training set increases as part of the iterative learning loops. We refer to the initially sampled and annotated training set as the ”initial training set”, and the remaining unannotated images from the patients in the training data as the ”unannotated train set”.

#### Ground truth generation

**Box annotation**: We used box annotations to differentiate between severely damaged regions and those that were moderately affected or unaffected by the disease. A region was considered severely affected by Stargardt disease where at least one retinal layer was undetectable. This area was enclosed within an orthogonal box drawn on the B-scan. If multiple small regions with undetectable layers were present across the retina, they were grouped together within a single larger box. For consistency, the enclosed area will be referred to as the ”severely affected region,” while the area outside the box will be termed the ”relatively unaffected region. It is important to note that the region outside the box may not be healthy-in fact, its ”health” is the very question that thickness measurement through segmentation aims to answer.

**Fig. 2:**
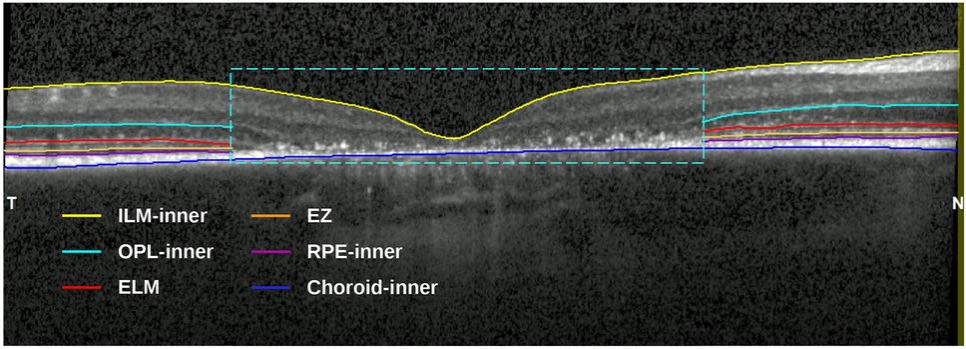
An example of an annotated B-scan with a box indicating the severely affected region, six boundaries outside the box, and two boundaries inside the box.

**Retina segmentation**: Outside the box, where retina sublayers were detectable, retina was manually segmented into five sublayers using the “OCTExplorer” software developed by the University of Iowa^28–30^. The sublayers are defined as follows (see Figure 2):

- Inner Retina (IR): from the Inner Limiting Membrane (ILM) to the inner boundary of the Outer Plexiform Layer (OPL).
- Outer Nuclear Layer (ONL): from the OPL inner boundary to the External Limiting Membrane (ELM).
- Photoreceptor Inner Segment (PR-IS): from the ELM to the posterior side of the Ellipsoid Zone (EZ).
- Photoreceptor outer segment (PR-OS): from the posterior EZ to the inner boundary of Retinal Pigment Epithelium (RPE).
- Retinal Pigment Epithelium (RPE): from the RPE inner boundary to the choroid inner boundary.

For the region inside the box, due to the complexity and degeneration of the retinal layers, only the total retinal thickness was segmented as a single layer.

Both annotation steps were performed by a trained individual and verified by a second trained reviewer. Any disagreements were resolved through discussion with an ophthalmologist.

### Image analysis

**Fig. 3:**
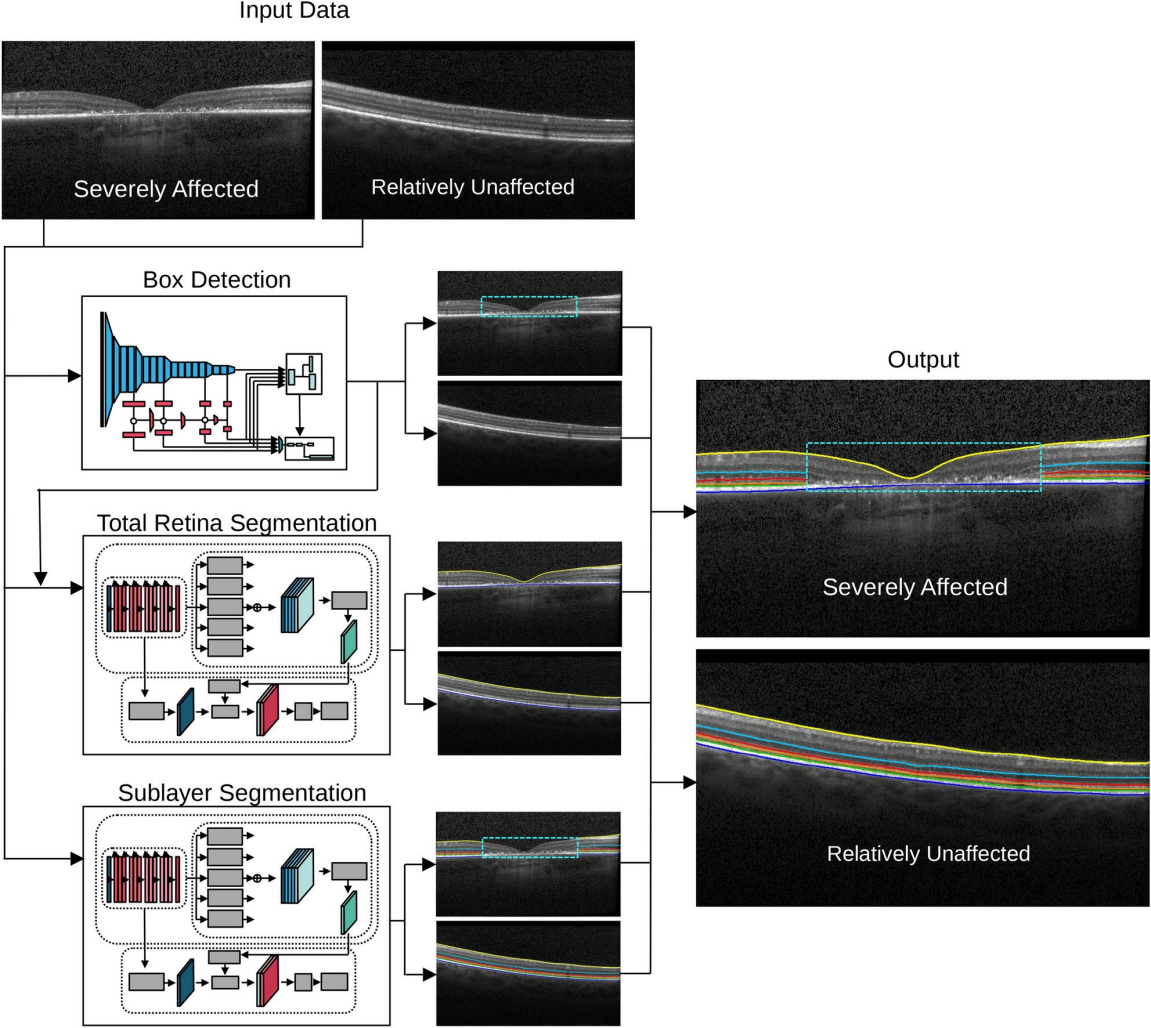
Our pathology-aware segmentation approach consists of three steps: 1) Box detection: A box detection model identifies severely affected regions in 2D B-scans, marking them with a rectangular box. 2) Total retina segmentation: A second deep learning model segments the total retina across the entire B-scan. 3) Sublayer segmentation: A second deep learning model segments retinal sublayers outside the box, with a loss function that excludes the severely affected region.

Our approach comprises three main steps (see Figure 3):

1. Detection of the severely affected region—box detection: Identifying the regions severely affected by Stargardt disease in 2D B-scans using a box detection model, marking these areas with a rectangular box. The box delineates severe damage that does not allow further segmentation other than total retina.
2. Segmentation of the total retina: Using a deep learning model to segment the total retina across the entire B-scan, focusing on the total retina, which remains visible in most Stargardt cases.
3. Segmentation of retinal sublayers: Applying a second deep learning model to segment retinal sublayers by integrating the information obtained from the first step. The loss function accounts for the severely affected region by excluding it from the calculation, thus focusing only on the relatively unaffected regions outside the box for sublayer segmentation.

Each model is integrated into an iterative learning algorithm, reducing the need for comprehensive data annotation, by acquiring manual annotation for only a sample of the data. The following sections describe the detailed implementation of the image analysis.

#### Iterative learning

To reduce the time-consuming and costly effort of labeling, we employed an iterative learning approach inspired by the active learning method^27,31^. Active learning involves iteratively selecting the most informative data from an unlabeled pool to improve model performance. The process begins with a labeled sample of the dataset. In the end of each loop, the trained model identifies the most informative subset of the unlabeled data, often based on uncertainty^32,33^. This new data is labeled and added to the training set, and the model is retrained. The process repeats until the model’s performance on the validation set stabilizes and no longer improves.

This approach was adopted by Block et al., who used a Monte Carlo Dropout method to identify the most uncertain images in an object detection task by calculating uncertainty based on the overlap between predicted bounding boxes^34^. However, this method assumes that every image contains at least one object. This assumption is unsuitable for our dataset, where some images may have no detectable objects (i.e. no Stargardt-affected region) but are still informative and valuable for training. To address this limitation, we modified this approach by employing a random sampling method to select data from the unlabeled pool, ensuring the inclusion of images with varying levels of degeneration. In the end of each learning loop, 200 images were randomly sampled from the unlabeled pool, labeled, and then added to the training set. The validation and test sets remained unchanged throughout the iterations.

#### Detection of the severely affected region—box detection

A Faster R-CNN-based model was trained to detect severely affected regions in 2D B-scans^35^. Specifically, we utilized the Faster R-CNN (R50-FPN) model from the Detectron2 platform developed by Facebook AI Research^36^, which incorporates a ResNet50^37^ backbone and a Feature Pyramid Network (FPN)^38^. The ResNet50 backbone was initialized with weights pre-trained on the ImageNet dataset^39^. The model was configured with a learning rate of 0.0005 and 2000 iterations for the first loop of the iterative learning. As the training set expanded, the number of iterations was increased throughout the iterative learning loops, reaching 3000 after 10 loops. We stopped the iterative training after 10 loops as the model’s performance reached a plateau (See Supplementary Materials).

The training, validation, and test sets used for box detection included only ProgStar data; no healthy images were considered in this phase. The model was built and executed within a Singularity container^40^ based on Ubuntu 20.04, customized to include Python 3.8, PyTorch 1.11.0, and CUDA 11.3.

#### Segmentation of the total retina

For the segmentation of the total retina across the entire B-scan, we employed the DeepLabv3 model^15^ with a ResNet50 backbone pre-trained on the ImageNet dataset^39^. The images were segmented into two classes: the total retina and the background. Each loop of the iterative training consisted of 15 epochs with a batch size of 15. The learning rate was set to 0.0005 at the beginning of each training loop. The adaptive moment estimation (ADAM) optimizer was used^41^. To optimize the Dice score on the validation set, a learning rate scheduler with a decay rate of 0.5 and a patience of 15 epochs was employed. The loss function was a combination of cross entropy loss $L_{ce}$ and the Dice loss $L_{dc}$ calculated as follows:

1 $$\begin{aligned} loss_{ce}= & -\frac{1}{N} \sum _{i=1}^{N} y_i \log (\hat{y}_i) \end{aligned}$$

2 $$\begin{aligned} loss_{dc}= & 1 - \frac{2 \sum _{i=1}^{N} y_i \hat{y}_i}{\sum _{i=1}^{N} y_i + \sum _{i=1}^{N} \hat{y}_i} \end{aligned}$$

3 $$\begin{aligned} loss= & loss_{ce} + loss_{dc} \end{aligned}$$

Here, $y_i$ is the ground truth label for the *i*-th pixel, $\hat{y}_i$ is the predicted probability for the *i*-th pixel, and *N* is the number of pixels in the entire batch of images.

Dice scores were evaluated on the validation set over five loops of training. At the end of each loop, 200 images were randomly sampled, labeled and added to the training set for the next loop. The training, validation and test sets included an equal share of ProgStar and healthy data.

The model was executed within a PyTorch NGC container (release 22.11) provided by NVIDIA, which included PyTorch version 1.13.0, CUDA 11.8, running on Ubuntu 20.04 with Python 3.8.

#### Segmentation of the retinal sublayers

For the regions outside the detected boxes, where retinal sublayers are visible, we used a procedure similar to that described for total retina segmentation, with some modifications described as follows. Here, we segmented five retinal sublayers—IR, ONL, PR-IS, PR-OS, and RPE—resulting in six classes, including the background. Given the increased complexity of this task, the number of epochs was increased to 30. Along with the 2D B-scans, the box information for the severely affected region, as detected in the box detection step, was provided as input to the model. The loss calculation was adjusted to exclude the severely affected region by using the box information as follows:

4 $$\begin{aligned} loss_{pa,ce}= & -\frac{1}{N} \sum _{i=1}^{N} m_i y_i \log {m_i \hat{y}_i} \end{aligned}$$

5 $$\begin{aligned} loss_{pa,dc}= & 1 - \frac{2 \sum _{i=1}^{N} m_i y_i\hat{y}_i}{\sum _{i=1}^{N} m_i y_i + \sum _{i=1}^{N} m_i \hat{y}_i} \end{aligned}$$

6 $$\begin{aligned} loss_{pa}= & loss_{pa,ce} + loss_{pa,dc} \end{aligned}$$

Here, $m_i$ is a mask with binary values, 0 for pixels inside the detected box and 1 for pixels outside the box. $loss_{pa,ce}$ is the pathology-aware cross entropy loss, $loss_{pa,dc}$ is the pathology-aware Dice loss, and $loss_{pa}$ is the pathology-aware total loss. To simplify, the mask height was extended to span the entire height of the image, ensuring that only pixels to the left and right of the box were considered in the loss. Masking the severely affected region in this study is analogous to the approach used by Wang et al.^21^, where unannotated regions were excluded from loss calculation.

We employed three models for sublayer segmentation: UNet^16^, DeepLabV3, and ReLayNet^42^. The UNet architecture was based on this Git repository^43^, with 16 filters. The ReLayNet architecture was borrowed from this Git repository^44^, using the same configuration. The DeepLabV3 arcitechture, as well as the learning rate and optimizer for all three models were the same as those used for total retina segmentation. The models were trained over five iterative loops. At the end of each loop, 200 randomly sampled and labeled images were added to the training set for the subsequent loop.

### Evaluation metrics

**Precision for box detection**: To evaluate the performance of the box detection model, the average precision (AP) was calculated as the area under the precision-recall (PR) curve. This curve was generated by plotting precision against recall, using a confidence threshold of 0.5 to determine whether a predicted bounding box was considered a valid detection. The precision and recall were defined as follows:

7 $$\begin{aligned} Precision= & \frac{True Positives}{True Positives + False Positives} \end{aligned}$$

8 $$\begin{aligned} Recall= & \frac{True Positives}{True Positives + False Negatives} \end{aligned}$$

The PR curve was obtained by varying the Intersection over Union (IoU) thresholds, which measure the overlap between the predicted bounding box and the ground truth bounding box. Two IoU thresholds of 50% (IoU = 0.5) and 75% (IoU = 0.75) were used to calculate the AP50 and AP75, respectively. The overall performance of the model was summarized using the mean Average Precision (mAP), which was the mean of the AP values across multiple IoU thresholds.

**Dice score**: To evaluate the performance of segmentation models, the Dice score, DC, was used. The Dice score measured the overlap between the predicted segmentation and the ground truth segmentation and was defined as twice the area of overlap between the predicted segmentation and the ground truth, divided by the total number of pixels in both areas:

9 $$\begin{aligned} DC = \frac{2 \sum _{i=1}^{N} y_i \hat{y}_i}{\sum _{i=1}^{N} y_i + \sum _{i=1}^{N} \hat{y}_i} \end{aligned}$$

The Dice Score ranges from 0 to 1, where a score of 1 indicates perfect overlap between the predicted and actual segmentation, while a score of 0 indicates no overlap.

## Results

### Detection of the severely affected region—box detection

**Fig. 4:**
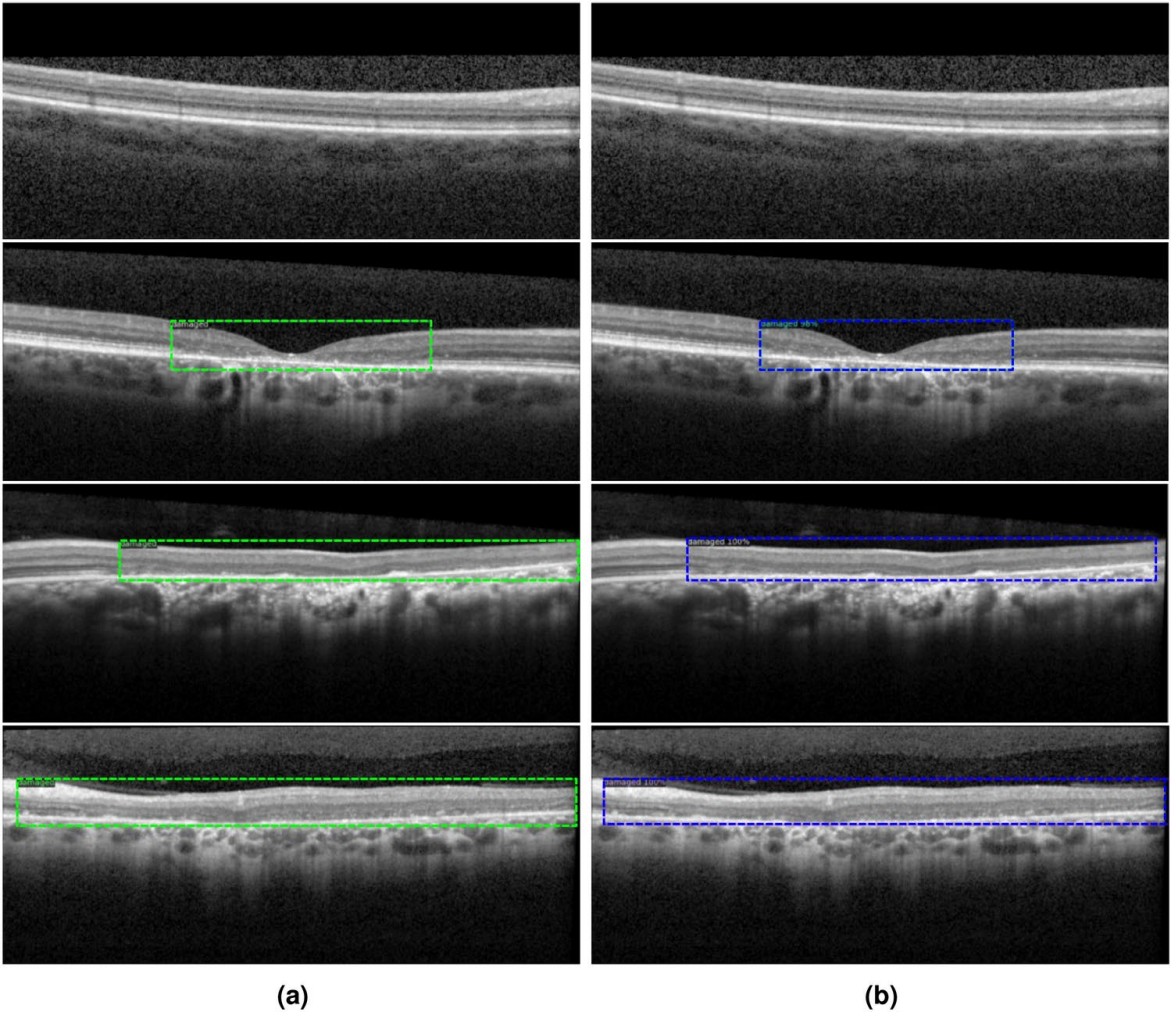
Examples of (**a**) ground truth and (**b**) prediction for the detection of the severely affected region (box detection).

Figure 4 shows several B-scans from different cross-sections with varying levels of damage, along with the ground truth and pred icted bounding boxes identifying the severely affected regions. In all test set images, the model successfully predicted the presence or absence of a bounding box. The precision of the predicted boxes was evaluated on the test set, yielding values of 65.31, 90.55 and 76.53 for mAP, AP50 and AP75, respectively. These results indicate that the model performed well in detecting the severely affected regions, particularly effective in identifying regions with a reasonable overlap (IoU threshold of $50\%$). Additionally, the AP75 value suggests that the model maintained strong performance even with more restrict overlap (IoU threshold of $75\%$). The mAP score of 65.31 reflects robust overall performance across multiple IoU thresholds, highlighting the model’s generalization ability.

### Segmentation of the total retina

The performance of the segmentation model for the total retina was assessed based on Dice score. The average Dice score across the entire test set was $99.1\%$. Specifically, the model achieved a Dice score of $99.3\%$ for B-scans of healthy eyes and $98.9\%$ for B-scans of patients with STGD1. These results indicate high accuracy in the segmentation of total retina, with slightly higher performance observed for healthy eyes compared to the affected by Stargardt disease.

It is important to note that the pathology-aware model, assigned for sublayer segmentation, was employed specifically for regions outside the box. Its performance for total retina segmentation was evaluated by aggregating sublayers for the regions outside the box on Progstar data. This approach yielded a lower Dice coefficient ($91\%$) compared to a dedicated single-layer total retina segmentation ($99\%$). Therefore, we opted for a distinct model for total retina segmentation, which directly segmented the retina as a single layer.

### Segmentation of the retinal sublayers

For the segmentation of the retinal sublayers, we compared the performance of our proposed method, which uses a pathology-aware loss function (Equation 6) with a standard model that employs a conventional loss function (Equation 3), using three network architectures for both approaches: UNet, DeepLabV3 and ReLayNet. The standard model was trained exclusively on the healthy dataset, as it was not feasible to use the ProgStar dataset due to the unavailable annotation for severely affected regions.

The models were evaluated on two test sets: one containing healthy data, and one containing ProgStar data. The performance of the segmentation models was assessed using the average Dice score across all retinal sublayers, as well as the average Dice score for each individual retinal sublayer. A summary of the results is presented in Table 1, comparing the performance of the standard approach and the pathology-aware approach across three models and two test sets.

**Table 1:** Dice scores evaluating the performance of the standard and pathology-aware methods across different test sets (Healthy and Progstar). The results are derived using three models (UNet, DeepLabv3, and ReLayNet) and are reported for individual retinal layers-Inner Retina (IR), Outer Nuclear Layer (ONL), Photoreceptor Inner Segment (PR-IS), Photoreceptor Outer Segment (PR-OS), and Retinal Pigment Epithelium (RPE)-as well as an overall score. Each score includes mean and standard deviation values.

Method	Test set	Model	Dice score					
			IR	ONL	PR-IS	PR-OS	RPE	Overall
Standard	Healthy	UNet	0.98±0.01	0.96±0.02	0.95±0.02	0.94±0.02	0.91±0.02	0.95±0.01
		DeepLabv3	0.98±0.01	0.96±0.02	0.94±0.02	0.94±0.02	0.91±0.02	0.95±0.01
		ReLayNet	0.98±0.01	0.96±0.02	0.95±0.02	0.94±0.01	0.91±0.02	0.95±0.01
	Progstar	UNet	0.86±0.31	0.80±0.28	0.79±0.20	0.80±0.23	0.81±0.28	0.81±0.21
		DeepLabv3	0.78±0.38	0.73±0.35	0.67±0.32	0.69±0.33	0.72±0.35	0.72±0.34
		ReLayNet	0.81±0.35	0.82±0.25	0.73±0.28	0.77±0.27	0.81±0.29	0.79±0.24
Pathology-aware	Healthy	UNet	0.98±0.01	0.96±0.02	0.95±0.02	0.94±0.01	0.91±0.02	0.95±0.01
		DeepLabv3	0.98±0.01	0.96±0.02	0.94±0.02	0.94±0.01	0.91±0.02	0.95±0.01
		ReLayNet	0.98±0.01	0.96±0.02	0.94±0.02	0.94±0.02	0.91±0.02	0.95±0.01
	Progstar	UNet	0.97±0.10	0.94±0.11	0.90±0.13	0.91±0.11	**0.92**±**0.10**	0.93±0.10
		DeepLabv3	0.92±0.23	0.91±0.19	0.89±0.16	0.89±0.16	0.89±0.18	0.90±0.15
		ReLayNet	**0.98**±**0.07**	**0.94**±**0.10**	**0.91**±**0.11**	**0.91**±**0.09**	0.92±0.11	**0.93**±**0.09**

**Fig. 5:**
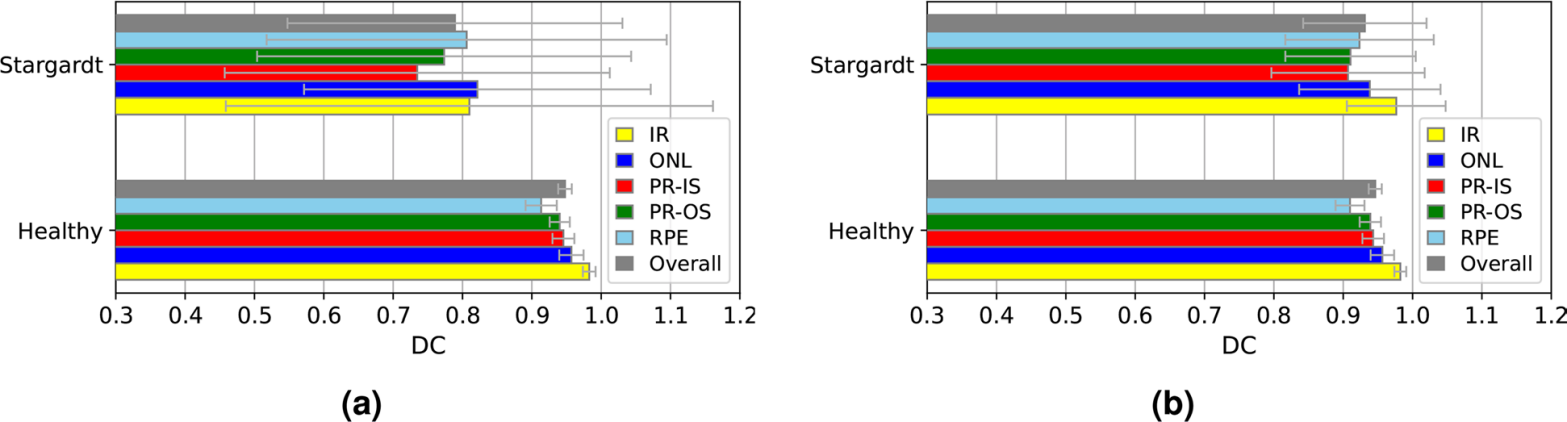
Dice scores, DC, for predictions using (**a**) the standard model and (**b**) the pathology-aware model on the Healthy and Stargardt datasets, using the ReLayNet model, evaluated for individual retinal sublayers and the overall Dice score across all sublayers. The bars represent the mean of Dice scores and the whiskers represent their standard deviations. IR: Inner Retina, ONL: Outer Nuclear Layer, PR-IS: Photoreceptor Inner Segment, PR-OS: Photoreceptor Outer Segment, RPE: Retinal Pigment Epithelium.

The plots depicted in Figure 5 visualize some of the Dice scores presented in Table 1, comparing the performance of the standard and pathology-aware methods across healthy and ProgStar datasets for the ReLayNet architecture. As the plots highlight this, both methods showed similar performance for the healthy dataset with an overall Dice coefficient of 0.95. For the ProgStar dataset, the pathology-aware model outperformed the standard model significantly (p < 0.001, paired T-test), achieving an overall Dice coefficient of 0.93 compared to 0.79 for the standard approach in the ReLayNet architecture.

The detailed breakdown of Dice coefficients for each retinal sublayer—IR, ONL, PR-IS, PR-OS, and RPE— shows that the pathology-aware method consistently outperformed the standard method for ProgStar data across all three network architectures, with improvements ranging from $13\%$ to $33\%$ for different sublayers. The largest improvement was observed in the PR-IS sublayer. Among the three architectures, ReLayNet showed the best performance, followed by UNet.

**Fig. 6:**
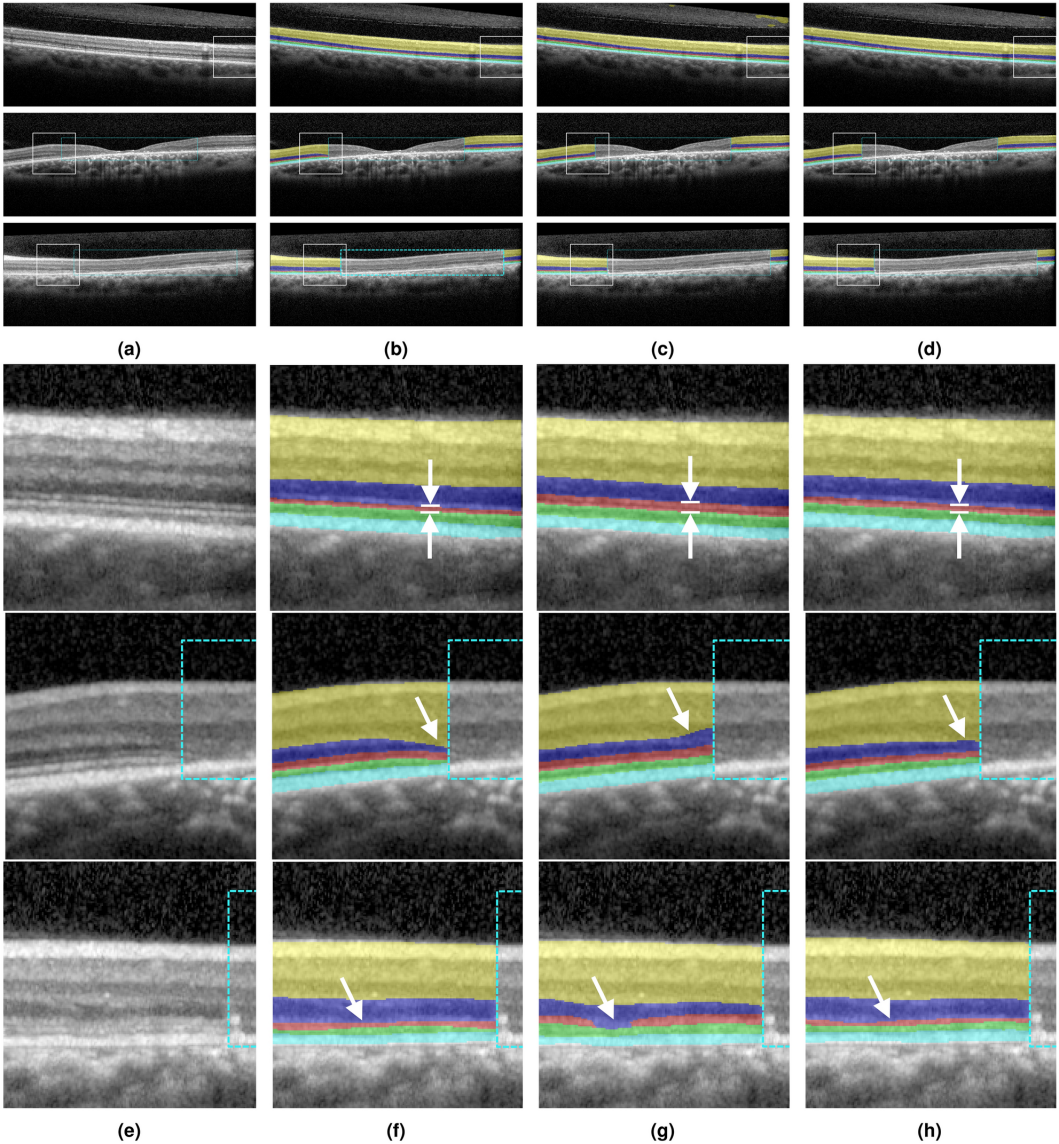
(**a**) B-scan image; (**b**) ground truth segmentation; (**c**) prediction using the standard method; (**d**) prediction using the pathology-aware method. (**e-h**) Corresponding zoomed-in views of (**a-d**), respectively. The retinal sublayers IR, ONL, PR-IS, PR-OS, and RPE are colored from top to bottom with yellow, dark blue, red, green, and cyan. The cyan box indicates the severely affected region where the segmentation is not performed. The white box shows the zoomed-in area. The arrows point to the areas where the standard method does not perform well.

Figure 6 presents example B-scans with segmented sublayers, comparing the ground truth with the predictions from both the standard method and the pathology-aware method.

## Discussion

In this study, we presented a novel deep learning approach for the automated segmentation of retinal sublayers in SD-OCT images from patients with STGD1. The key contribution of our method lies in the introduction of a pathology-aware loss function, which enables the model to focus on relatively unaffected regions of the retina while adapting to the variability in retinal degeneration across different patients.

The use of box detection in our study in severely affected regions avoids the challenge of attempting to segment absent layers. In these areas, segmenting the retina as a single layer provides a more reliable and feasible approach, ensuring that the segmentation task remains manageable and clinically relevant. Attempting to segment individual layers in such regions would not only introduce inaccuracies but also complicate the interpretation of the results. By focusing on relatively intact regions, the method captures detailed information where possible, while maintaining robustness in areas with advanced degeneration.

Retinal degeneration can vary widely from person to person, with some regions of the retina being more affected than others. The method can recognize and handle these differences, focusing on the relatively unaffected regions of the retina in each individual case, even though the extent and nature of the degeneration may differ across patients and visits. Our results demonstrate that the proposed method achieved high performance in segmenting retinal sublayers in less affected regions and the total retina in severely affected regions. This dual approach enhances the model’s ability to generalize across a diverse dataset with varying degrees of retinal degeneration.

The iterative learning strategy employed in our study was particularly effective in reducing the annotation burden, which is a critical factor for the scalability of such techniques in larger clinical studies. By iteratively sampling new images, the models were able to improve their performance with a relatively small labeled dataset; $\sim 1700$ images equivalent to $\sim 1.5\%$ of the total ProgStar dataset for box detection, and $\sim 1000$ images equivalent to $\sim 1\%$ of the total ProgStar dataset for the segmentation tasks. This approach is highly relevant for clinical applications where obtaining large, annotated datasets is often challenging. Monitoring the model performance on the validation dataset over consecutive loops ensured that we could take the most out of data without losing much information by not including the whole dataset.

Compared to previous approaches, our method offers several key improvements. Earlier studies either focused on segmenting the total retina as a single layer across the entire image^11,25^, or complicated the segmentation task by attempting to segment an excessive number of sublayers across the entire B-scan, including regions with severe disruption^26^. The former lacked the granularity needed to better understand the disease, while the latter made the segmentation task overly complex with the risk of losing reproducibility in regions with significant degeneration. Our method achieves an optimal balance, extracting as much detailed information as possible by introducing a pathology-aware loss function that differentiates between severely affected and relatively unaffected regions. This gives us the possibility to investigate the layers that are still visible, although have already undergone some degeneration. Additionally, the method remains straightforward enough to be integrated into clinical practice.

The ability to accurately segment retinal sublayers in OCT images has major implications for the clinical management of Stargardt disease. Visual acuity, while clinically important, is not a good outcome measure, because the disease is characterized by a progressive enlargement of visual field loss while only the foveal center drives visual acuity. In addition, visual acuity is a subjective and inherently variable measure, which limits its reliability. Structural measures such as OCT imaging, equipped with precise quantification of retinal layer thicknesses and the extent of atrophy can provide valuable biomarkers for tracking disease progression and assessing the efficacy of potential therapies. The automated approach presented here could facilitate more consistent measurements, and enable large-scale clinical trials.

Moreover, our method’s capacity to handle data with varying degrees of retinal damage makes it particularly suitable for longitudinal studies where the extent of degeneration changes over time. By focusing on relatively unaffected regions for detailed analysis, while still capturing overall retinal thickness in affected areas, our approach can offer a more comprehensive assessment of disease progression.

It is important to note that OCT images do not provide an immediate reflection of the patient’s vision, and the ongoing debate concerns whether OCT imaging can serve as a reliable surrogate endpoint. Therefore, our study could be further expanded to explore the structure/function relationship in Stargardt disease. By integrating functional measures such as microperimetry with OCT-derived structural data, future studies could investigate how retinal degeneration correlates with functional vision loss. Furthermore, integrating other imaging modalities, such as fundus autofluorescence, could provide complementary information to enhance box detection and segmentation accuracy.

In addition to thickness analysis, the size of the detected boxes could be utilized in future studies to monitor the growth of the damaged area over time, offering a new dimension to the longitudinal analysis.

Further refinement of the iterative learning strategy, moving from a random selection to selecting more informative data at successive loops, possibly by incorporating uncertainty estimation methods, could improve the efficiency and effectiveness of the annotation process.

Our current approach effectively detects single boxes including the affected region. However, in some cases, there are still detectable boundaries inside the box which are not considered due to the single box strategy, as we combined multiple small regions into one large box. These boundaries might hold critical information, and refining our approach to implement a multi-box detection mechanism could enhance the analysis by adding more details from such regions which are covered by the larger boxes.

Another limitation of the current study is the use of data from a single type of OCT device, specifically the Heidelberg Engineering system. To ensure the reliability of our approach across a broader range of clinical settings, further evaluation using datasets from other OCT scanners and expanding the test set with external datasets would enhance the applicability of the results across diverse clinical environments. Additionally, while the pathology-aware approach requires relatively less complex annotations, due to the exclusion of severely affected regions, the reproducibility of these annotations could be assessed through blind grading by multiple graders.

Our method is model-independent and can utilize any deep learning model for box detection and segmentation. Although the results show a significant accuracy in the prediction, other deep learning models, such as transformers^45^ or dual head convolutional networks^46^ which combine boundary regression with layer segmentation could be investigated for better performance in segmentation.

Although our method is tailored for STGD1, its versatility allows it to be adapted for other diseases, such as geographic atrophy in age-related macular degeneration, or imaging modalities where segmentation of organs with damaged tissue structures is required.

## Conclusion

Our study introduces a novel, pathology-aware deep learning approach for the automated segmentation of retinal layers in SD-OCT images of patients with STGD1. By focusing on relatively unaffected regions and adapting to the variability in retinal degeneration, our method provides a balanced and efficient means of extracting valuable structural information while maintaining simplicity for clinical implementation. The integration of an iterative learning strategy significantly reduces the annotation burden, making the approach scalable for larger studies. This work not only advances the field of automated OCT segmentation but also lays the foundation for future research aimed at enhancing the understanding and clinical management of Stargardt disease and related retinal disorders.

## Supplementary Information

Supplementary Information.

## Data Availability

The datasets generated and/or analysed during the current study are not publicly available due to the inclusion of clinical data but are available from the corresponding author on reasonable request.
