# Supplementary material for: Looking outside the box with a pathology aware AI approach for analyzing OCT retinal images in Stargardt disease
Source: Sci Rep. 2025 Feb 8;15:4739. doi: 10.1038/s41598-025-85213-w (PMC11807158; doi:10.1038/s41598-025-85213-w)
Supplement: Supplementary file 1 — Supplementary Information. [file 41598_2025_85213_MOESM1_ESM.pdf]

# Supplementary Material

## Box Detection—Iterative Training Details

Figure S1 illustrates the performance of the box detection model in terms of precision values on the validation set, showing that performance reached a plateau over the course of 10 loops.

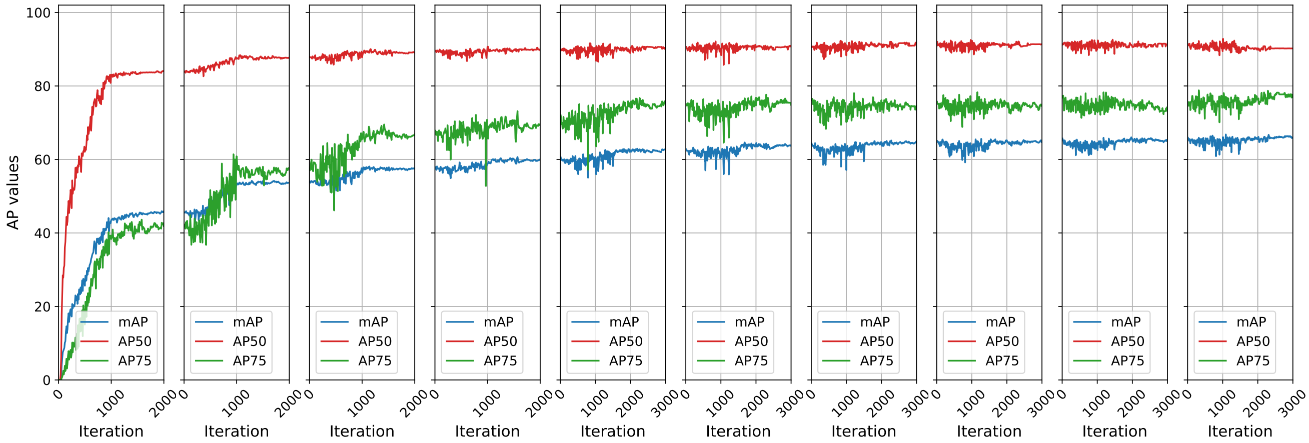


**Figure S1.** Precision values for the validation set for the box detection model over 10 loops of training. The size of the training set grows with steps of 200 images at each loop.

## Segmentation—Iterative Training Details

For the segmentation of total retina and retinal sublayers, Dice scores were evaluated on the validation set over five loops of training. The initial performance, for the total retina segmentation, after the first two loops was already very high (> 0.99%), and the subsequent gains were marginal. Therefore, we stopped the training after five loops.

Figure S2 presents Dice scores assessed on the validation set across five training loops for the segmentation of retinal sublayers using the DeepLabV3 model. There is only a marginal improvement after the third loop. Consequently, training was halted after five loops.

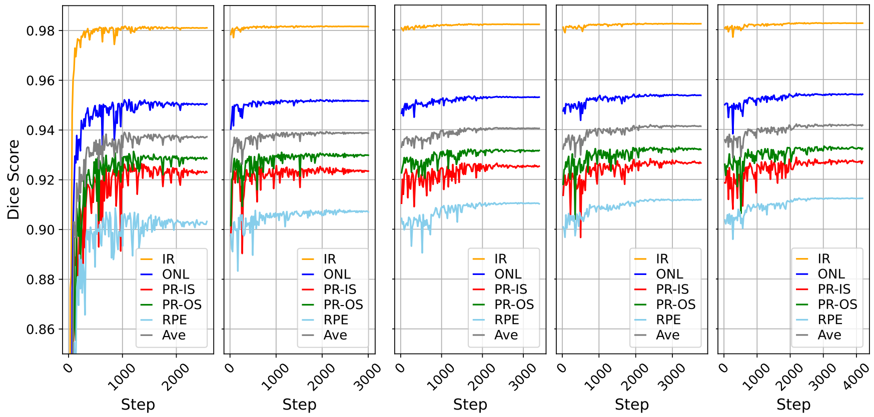


**Figure S2.** Dice scores, DC, for the retinal sublayers segmentation assessed on the validation dataset over five loops of training. The size of the training set grows with steps of 200 images at each loop. Steps indicate the weight updates based on one batch of images.
